# Concordance of gene expression in human protein complexes reveals tissue specificity and pathology

**DOI:** 10.1093/nar/gkt661

**Published:** 2013-08-05

**Authors:** Daniela Börnigen, Tune H. Pers, Lieven Thorrez, Curtis Huttenhower, Yves Moreau, Søren Brunak

**Affiliations:** ^1^Department of Electrical Engineering, ESAT-SCD, IBBT-KU Leuven Future Health Department, KU Leuven, 3001 Leuven, Belgium, ^2^Biostatistics Department, Harvard School of Public Health, Harvard University, Boston, 02115 MA, USA, ^3^Broad Institute of MIT and Harvard, Cambridge, 02142 MA, USA, ^4^Department of Systems Biology, Center for Biological Sequence Analysis, Technical University of Denmark, DK-2800 Lyngby, Denmark, ^5^Program in Medical and Population Genetics, Broad Institute of MIT and Harvard, Boston, 02142 MA, USA, ^6^Division of Endocrinology and Center for Basic and Translational Obesity Research, Children’s Hospital Boston, Boston, 02115 MA, USA, ^7^Department of Development and Regeneration @ Kulak, KU Leuven, E. Sabbelaan 53, 8500 Kortrijk, Belgium, and ^8^NNF Center for Protein Research, Health Sciences Faculty, University of Copenhagen, DK-2200 Copenhagen, Denmark

## Abstract

Disease-causing variants in human genes usually lead to phenotypes specific to only a few tissues. Here, we present a method for predicting tissue specificity based on quantitative deregulation of protein complexes. The underlying assumption is that the degree of coordinated expression among proteins in a complex within a given tissue may pinpoint tissues that will be affected by a mutation in the complex and coordinated expression may reveal the complex to be active in the tissue. We identified known disease genes and their protein complex partners in a high-quality human interactome. Each susceptibility gene's tissue involvement was ranked based on coordinated expression with its interaction partners in a non-disease global map of human tissue-specific expression. The approach demonstrated high overall area under the curve (0.78) and was very successfully benchmarked against a random model and an approach not using protein complexes. This was illustrated by correct tissue predictions for three case studies on leptin, insulin-like-growth-factor 2 and the inhibitor of *NF-κB* kinase subunit gamma that show high concordant expression in biologically relevant tissues. Our method identifies novel gene-phenotype associations in human diseases and predicts the tissues where associated phenotypic effects may arise.

## INTRODUCTION

Although most genes and their protein products function as modules within biological networks, their tissue-specific functions in metazoans have largely been analysed from the perspective of individual disease genes. Previous studies have, for example, quantified the expression of >6000 proteins in individual tissues (1), and although these data typically target individual proteins, they can be linked with gene expression data to begin to analyse the disease-specific functions of entire complexes. Likewise, it is clear that phenotypes arising from mutations in human genes are usually highly specific to a limited number of tissues (2,3). A more integrative and systems-level approach to the problem of assigning tissue specificity to groups of disease-linked genes and proteins is therefore in order.

Recently, a strategy was developed for systematically correlating the manifestations of diseases with expression patterns of genes and protein complexes across human tissues (1). This established a significant tendency for disease genes to be over-expressed in tissues where defects cause pathology, even under non-disease conditions. This analysis was based on expression data from the GNF tissue atlas (4), delivering tissue-specific expression data for 73 normal tissues. More than 1000 diseases derived from Online Mendelian Inheritance in Man (OMIM) were analysed by integrating expression data with disease protein complexes. A second recent study integrated interaction and expression data to analyse the interplay between protein expression and physical interactions in human tissues (5). This revealed that most tissue-specific proteins normally interact with core cellular components, and that most universally expressed or ‘housekeeping’ proteins have tissue-specific protein interactions. Finally, a third examination of the dynamic structure of the interactome found changes in its organization predictive of breast cancer outcome (6). Specifically, intermodular hub proteins were found with low correlation with interaction partners and tissue-specific expression, in contrast with intramodular hub proteins with correlated patterns of co-expression across tissues. These were based on average Pearson correlation coefficients (PCCs) of co-expression of a hub protein and its partners to quantify context-specific interactions (that is, interacting proteins *not* consistently co-expressed) or constitutive (interacting proteins frequently co-expressed).

Considering these three studies together, Bossi and Lehner (2009) analysed the interplay between protein expression and physical interactions in humans, but without linking these to diseases (5). In contrast, Lage et al. (2008) correlated diseases with normal expression patterns of protein complexes across human tissues, but did not investigate protein complex co-expression (1). Finally, Taylor *et al.* (2009) identified changes in the organization of the interactome based on a co-expression measure and used these changes to predict breast cancer outcome (6). This work combines the strengths of these previous studies and builds on them to determine the tissue and disease specificity of a broad set of human protein complexes in a novel manner.

Here, we introduce TissueRanker, a predictive method for disease/tissue associations based on a co-expression measure of transcripts within human protein complexes, leveraging a recently published global map of human gene expression data (7). This larger set of human expression data allowed the analysis of disease-linked protein complexes specific to many tissues. In particular, TissueRanker uses the assumption that a protein complex in which the hub protein is confirmed to be involved in a certain disease should be well coordinated in expression in the normal tissue where mutations cause a disease phenotype. Accordingly, coordinated expression of disease genes with their protein interaction partners proved to provide direct insight into the tissues they affected in the disease. We demonstrated this using a benchmark dataset consisting of 248 gene-disease–tissue associations, resulting in accurate performance of area under the curve (AUC = 0.78) over all tissues (Figure 2B). This tissue ranking based on coordinated protein complex expression was more accurate than tests using individual proteins (AUC = 0.59), or a random model (AUC = 0.52). Three case studies were also illustrative: the leptin (*LEP*) gene recently linked to neurodegenerative disorders (8,9), which showed concordant expression in distinct brain tissues; the insulin-like growth factor 2 (*IGF2*), with concordant expression in smooth muscle and brain tissue, highlighting its biological roles in muscle differentiation and cerebellum morphology as found in previous studies (10,11); and the inhibitor of *NF-κB* kinase subunit gamma (*IKK-γ*), found to be co-expressed in prostate tissue as supported by previous findings (12,13).

## MATERIALS AND METHODS

**Figure 1.**
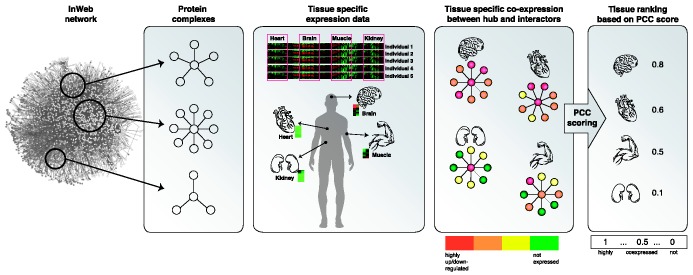
The key steps in the approach: First, for each candidate hub protein, we identify interaction partners from a human interactome and associate expression levels to each partner. Second, we compute for each protein complex its coordinated expression in all tissues by calculating the average co-expression (PCC) between the hub protein and its interaction partners. Third, we rank for each complex the tissues by their coordinated expression values (PCC scores). According to the underlying hypothesis, the tissue with the highest coordinated expression value will be the one where mutations would most likely cause a disease phenotype.

The key elements in TissueRanker are to compute a (normalized) coordinated expression value for each protein complex in each tissue by calculating the average co-expression PCC between a hub protein and its interaction partners in a provided underlying protein network. Tissues are subsequently ranked for each protein complex by their coordinated expression values (Figure 1). According to our hypothesis, the tissue with the highest coordinated expression value will be the one where deregulation of the hub gene most likely would cause a detrimental phenotype.

### Network of protein complexes

Protein–protein interaction networks are regarded as a reliable type of molecular interactomes as shown in (14). Protein–protein interactions give rise to a plethora of higher-order cellular structures (in this work referred to as protein complexes) that can act as molecular machines, such as the RNA polymerase 2 complex, form dynamic signaling networks, such as the JAK-STAT signaling pathway, or assemble into relatively rigid cellular structures, such as the cytoskeleton. To construct a set of high-confidence protein complexes, we used the InWeb v4.1 protein–protein interaction database that currently consists of 13 000 proteins and 158 201 protein interactions for extraction of 9889 protein complexes (14). We constructed protein–protein interaction subnetworks (referred to as protein complexes hereafter) by mapping the directly interacting proteins to a given hub protein (for all proteins having at least five interactor proteins), which resulted in 3590 protein complexes. A degree cutoff of >5 has been suggested to represent the highest 15% of the degree distribution of hub proteins (6). Protein can be present in several protein complexes.

### Global map of human gene expression data

We used a global map of human gene expression (7) containing 5372 samples and representing 128 different tissues in four different cell types (normal, disease, neoplasm and cell line). Among the tissues, 64 with normal status were available with up to 416 samples per tissue. Considering only experiments with at least five replicates (6) and excluding tissues that were not suitable for our analysis (such as lung tissue from transplants, current and former smokers, as well as a human universal reference), we ended up using expression data from 36 different tissues (Supplementary Table S1).

### Coordinated expression in protein complexes

For each hub protein, the average PCC of co-expression for each interaction and the hub was assessed using an algorithm similar to what was previously described (6, 15). Let a protein complex *C* consist of a hub protein *H* and its interactor 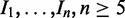
*.* Let 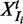 be the expression data of interactor 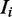 of hub *H* for a specific tissue *t*, and let 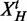 be the expression data for hub *H* for a specific tissue *t.* The PCC of co-expression, 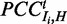, between a hub *H* and its interactor 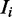 in a tissue *t* across all samples *j* can be computed by:
(1)
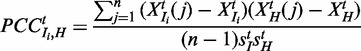



To compute the correlation between a hub protein *H* and all its interactors 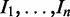 in complex *C*, the co-expression 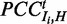 for each hub-interactor pair is computed. Taking the average of these single co-expression values results in the ‘coordinated expression’ of complex *C* for a specific tissue *t*:
(2)
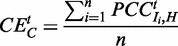



For each of the 3590 protein complexes, we computed the degree of coordinated expression of members in each complex across all tissues. This resulted in a 3590 × 36 coordinated expression matrix containing the coordinated expression for each protein complex in the underlying InWeb network across all 36 tissues.

### Statistical inference and tissue ranking

The number of samples highly varied among the tissues (Supplementary Table S1). To alleviate the potential bias, we have first applied the Fisher transformation to obtain approximately normal distribution (see Equation 3), and then standardized by computing standard scores (*z*-values):
(3)
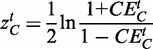

(4)
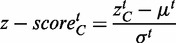



To determine in which tissue a given protein complex is most coordinated, we ranked the coordinated expression values of all tissues in decreasing order. We hypothesize that tissues with high coordinated expression values are the ones where genetic variation (or aberrant protein modification) in the corresponding hub protein most likely will cause detrimental downstream phenotypes.

### Gold standard for validation

To benchmark our approach, we compiled a gold standard set of gene–disease relationships. This was generated based on the disease–tissue association matrix defined in (1) combined with known disease–gene associations from OMIM (version February 2010) by assigning diseases from OMIM and corresponding tissues from (1) to each protein and its complex. Integration of these two datasets resulted in a gold standard matrix compiling 248 genes associated with 36 non-disease tissues and 165 distinct OMIM diseases (Supplementary Table S3). These relationships comprised our literature-based reference associating Mendelian disease genes with tissues and were used to benchmark performance of tissue predictions identified by TissueRanker.

### Defining tissue categories for validation

For comparison of tissues, we grouped the 36 tissues from our coordinated expression matrix (excluding bone tissue, as it was not present in the gold standard) and the 51 tissues from the gold standard into 14 tissue categories (Supplementary Table S2). Alternatively, our analysis would have been restricted to fewer tissues, namely those that are present in both datasets, thereby missing several subtissues.

These tissue groups are hence referred to as tissue categories. Most tissue categories comprise several tissues (e.g. the tissue category ‘heart’ contains ‘atrial myocardium’ and ‘cardiac ventricle’ in our approach, whereas it contains the tissues ‘cardiac myocytes’ and ‘atrioventricular node’ in the gold standard). To keep consistency and facilitate the tissue comparison, we refer to the tissue category whenever referring to a specific (sub-) tissue of a tissue (e.g. instead of using the specific tissue ‘cardiac myocytes’, we will term it by its category ‘heart’).

### Validation

To validate our predictions, we used the values from the pre-computed ‘coordinated expression matrix’ (as described earlier) for the 248 protein complex–disease associations across all tissues. We then ranked the tissues for each of these gene products according to their coordinated expression. We compared these predicted ranks with the ranks as defined by the gold standard. For some protein complexes, multiple distinct diseases were available [e.g. ATM is associated both with Breast Cancer, OMIM #114480 (16), and with Ataxia, OMIM #208900 (17)]. Because the gold standard ranks tissues according to their associations to diseases based on literature, and TissueRanker ranks tissues of protein complexes according to their coordinated expression in tissues, the gold standard produces distinct tissue rankings for the same protein as long as the disease is different. TissueRanker, however, produces a unique tissue ranking per protein complex, independent of its known disease association. Next, we used receiver operating statistics curves and AUC values to quantify sensitivity and specificity values of our predicted ranking compared with the gold standard ranking. This was achieved by turning the rankers into binary classifiers applying a threshold on the ranking that created a point in the receiver operating statistics curves space.

In addition to this performance assessment comparing the global TissueRanker predictions with the gold standard, we also reported the overlap of the top three predicted with the top three gold standard tissues: *r_GS_^m^_∩_ r_Pred_^m^* (for m = 1,..,3 if top three tissues are considered)*.*

To assess whether TissueRanker produced more accurate tissue predictions than single-protein expression analysis, we generated a tissue ranking for each protein in our benchmark by computing the test statistic of an unpaired** t-test between the expression values of one tissue and the expression values of the remaining tissues. The resulting tissue-specific test statistics for all tissues and all benchmark proteins were compared against the gold standard ranking in the same way as above.

Finally, to show that TissueRanker performs better than random expectations, we repeated our entire approach based on randomized gene expression data. Randomization was accomplished by shuffling genes tissue-specific expression values for each tissue 100 times, followed by recomputing coordinated expression for each protein complex.

### Tissue distance matrices

Based on the genome-wide expression data, we computed the pairwise correlation (PCC) between tissues, producing tissue distance matrices containing all the pairwise distances for a given set of tissues. Supplementary Figure S1 illustrates the distance matrices as heatmaps of all the 36 tissues from our study and the 14 benchmark tissue categories, respectively. From the tissue distance matrices, we applied the neighbor-joining algorithm (18) to generate unrooted distance trees displaying tissue-relationships in the space of gene expression levels.

## RESULTS

**Figure 2.**
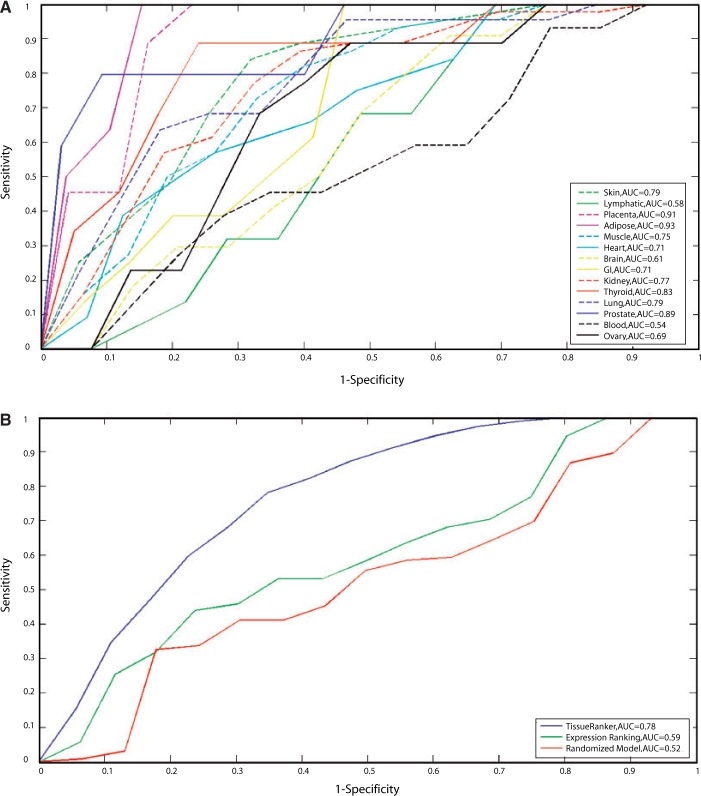
Receiver operating statistics curve (ROC) and corresponding AUC showing the performance of the tissue ranking in comparison with the gold standard for all 248 protein–disease associations from the benchmark (Supplementary Table S3). Curves are shown for each tissue separately in (**A**) and in total in (**B**).

We used 3590 protein complexes and a recently published global map of human gene expression data in TissueRanker to predict the tissue specificity of disease phenotypes arising from defects in these complexes. The approach was benchmarked using a gold standard set containing 248 unique human protein–disease associations (see ‘Materials and Methods’ section), resulting in an overall performance of an AUC of 0.78 across all tissues (Figure 2B). Although it is well established that genome-wide expression profiles can be used to quantify inter-tissue relationships (1,7), this method makes it possible to also determine the tissue specificity of much smaller transcriptional units, such as individual pathways or protein complexes. Details of the method, its validation and several biological examples are as follows.

### Global performance of TissueRanker across human tissues

**Figure 3.**
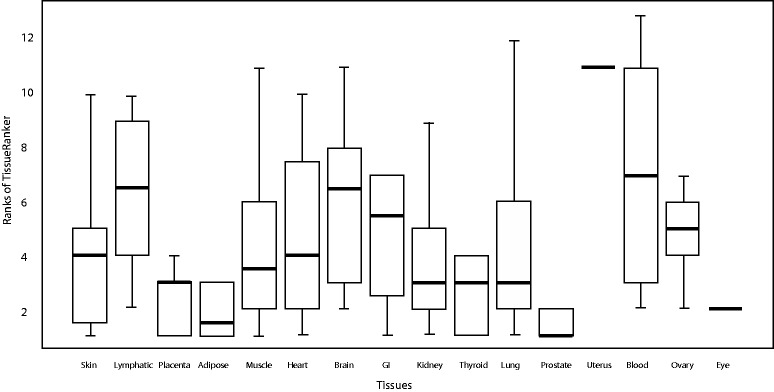
Distribution of the tissue ranks showing the performance of the tissue ranking in comparison with the gold standard for all 248 protein-disease associations from our benchmark (Supplementary Table S3) for each tissue separately. Note that only either one or two tissues were ranked in the top for eye and uterus, respectively. Therefore, no boxplots are shown here for these two tissues.

To assess TissueRanker's accuracy of prediction within individual tissue contexts, we benchmarked our approach against a literature-derived gold standard set, achieving AUC values >0.8 for most tissues (Figure 2A). Average performance across all individual tissues was AUC = 0.75, but as expected, this varied by tissue type. The best performing tissues (i.e. most accurately predicted disease associations) were adipose (0.94), placental (0.91), prostate (0.89) and thyroid (0.83), whereas the worst tissues were blood (0.54), lymphatic system (0.58), brain (0.61) and ovary (0.69). The broadest distribution of ranks was observed in blood (see Figure 3), possibly owing to the large number of heterogeneous samples (437) included in the ‘tissue’ (see Supplementary Table S1), whereas the smallest distribution can be observed in the well-performing tissues, adipose, placenta and prostate (Figure 2A, Supplementary Figure S3). Thus, tissues that were more difficult to predict were characterized by a high diversity of included cell types (e.g. blood, lymphatic system or brain tissue), whereas well-characterized tissues were typically more homogeneous.

We next compared tissue predictions from our method with the gold standard by identifying the top three ranked tissues in each (see ‘Materials and Methods’ section). The benchmark set includes several proteins (40 of 243, 17%) that occur multiple times, as they are involved in multiple diseases [e.g. lamin A/C (LMNA**) is associated with all the three diseases, cardiomyopathy (19), Charcot-Marie-Tooth disease (20) and muscular dystrophy (21)]; additionally, some diseases are also associated with multiple proteins [e.g. diabetes is associated with 12 distinct proteins (Table 1)]. Therefore, we refer to 248 unique protein–disease associations in our benchmark set for proteins or diseases. Supplementary Figures S4 and S5 show that neither the size of the protein complex (number of proteins in the complex) nor the number of diseases in which a protein is involved had an impact on the performance (AUC) of its corresponding protein complex. This suggests that TissueRanker can identify multiple biologically meaningful tissues that may cause phenotypes in different diseases, independent of the size of the protein complex.

**Table 1.** The top ranked tissues (TissueRanker versus gold standard) for two example studies in diabetes mellitus type 2 and Leigh’s disease

Proteins	Ranking TissueRanker
Diabetes mellitus type 2 (OMIM #125853): muscle, adipose, ovary, brain, heart and kidney tissue	
ABCC8	prostate|lung|*kidney*
HNF4A	placenta|*heart*|prostate
IRS1	*adipose*|*brain*|skin
LIPC	placenta|*kidney*|blood
MAPK8IP1	placenta|prostate|*kidney*
NEUROD1	*ovary*|*muscle*|prostate
HNF1A	placenta|*heart*|*brain*
GCK	uterus|*kidney*|*adipose*
IGF2BP2	thyroid|placenta|skin
TCF7L2	placenta|*kidney*|blood
IL6	placenta|GI|*muscle*
PPARG	lung|placenta|*adipose*
Leigh’s disease (OMIM: #256000): Brain and muscle tissue	
DLD	heart|kidney|*brain*
NDUFS4	blood|*brain*|kidney
NDUFV1	blood|uterus|kidney
SDHA	uterus|blood|GI
NDUFA2	uterus|kidney|*muscle*

This table was extracted from the larger Supplementary Table S3.

Finally, we compared the performance of TissueRanker with a ranking based only on single-protein expression analysis. Specifically, we evaluated a tissue ranking obtained by computing each protein/tissue pair's *t*-statistic of that tissue's expression distribution compared with the pooled distribution across all other tissues (see ‘Materials and Methods’ section). This ranking (Figure 2B), again compared with the gold standard, also demonstrated that TissueRanker produced a more accurate tissue ranking based on coordinated expression within a protein complex (AUC = 0.78) than a simpler ranking based only on per-protein tissue-specific expression patterns (AUC = 0.59). To evaluate the performance of TissueRanker, we ran our approach based on randomized input data (see ‘Materials and Methods’ section) and observed that TissueRanker, as expected, performed better than random expectations (AUC = 0.52) (Figure 2B and Supplementary Figure S2).

### Assessing tissue similarity by global gene co-expression

**Figure 4.**
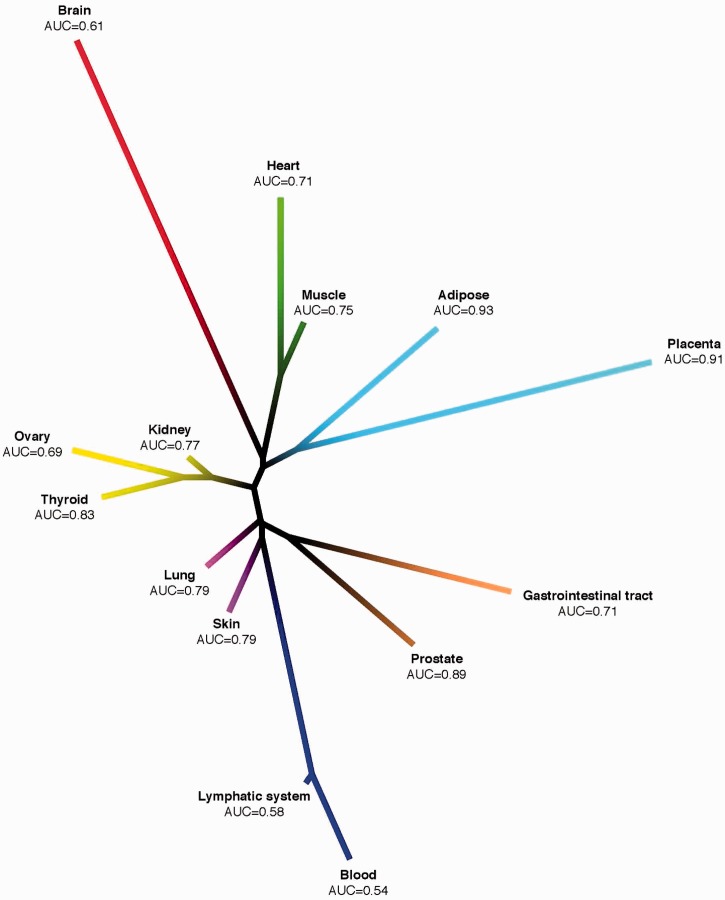
Unrooted tissue distance tree of the 36 tissues from our study based on pairwise correlation of global expression data.

**Figure 5.**
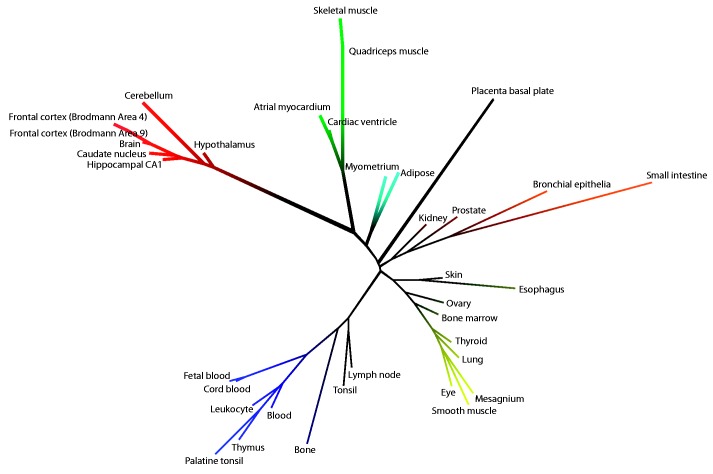
Unrooted tissue distance tree of the 14 tissue categories from our benchmark study based on pairwise correlation of global expression data.

This analysis also permitted a novel, expression-based, comparative genome-wide analysis of 36 human tissues (Figure 4). Different subtissue types (e.g. brain tissues and muscle tissues) have a clear tendency to cluster together, signifying that similar tissue types have similar transcriptional programs executed across the different cell types they contain. We have used the same approach to quantitatively evaluate our results across tissue categories, including a distance tree for the 14 tissue categories from our benchmark study with AUC values as described earlier in the text (Figure 5; Supplementary Figure S1B illustrates the corresponding heatmap). Tissues with similar co-expression patterns (Figure 4) also, in general, perform similarly in terms of disease predictability (Figure 5). For example, adipose and placenta cluster together and belong both to the top performing tissues with AUCs of 0.91 and 0.93, whereas heart and muscle obtained 0.71 and 0.75 as AUC values, as these tissues are close based on their pairwise correlation.

### Predictive performance within and across protein complexes

Next, we computed AUC values for each protein complex in our benchmark study separately, based on the comparison between our predictive tissue ranking and the gold standard ranking. Supplementary Figure S2 and Supplementary Table S5 show the resulting right-skewed distribution of all 248 complex-specific AUC values, demonstrating that our prediction introduces far less false-positive predictions than expected by chance.

Among the protein complexes that reached the highest AUC values (>0.9), we identified the TGFB1, SOD1 and the AVPR2 complexes (Supplementary Table S5). Tissues supported both by existing literature (i.e. the gold standard) and by consistent co-expression within the complex could be of particular interest for biological follow-up to better understand mechanism of disease or potential points of therapeutic intervention. On the lower side of the distribution, the IKBKG protein complex had the lowest AUC value (0.62), still well above background. Low-performing complexes were again characterized by high variability, in particular a greater diversity of phenotypes in which they were involved. Other examples included PTPN22 (AUC = 0.63), associated with two diseases in our benchmark displaying diverse phenotypes (rheumatoid arthritis, OMIM #180300, and systemic lupus erythematosus, OMIM #152700). SDHB, FGFR2 and MUTYH (AUC = 0.68, 0.67 and 0.65, respectively) were similarly associated with diverse cancer types and thus highly variable phenotypes (Supplementary Table S3). These protein complexes were generally associated with only one disease in the literature (Supplementary Table S3), however, and true predictive performance may be difficult to estimate because of underannotation.

In particular, specific evidence for other examples suggested the gold standard to be substantially under-annotated (e.g. for pleiotropic features), as for the IKBKG protein complex. The gold standard associates the IKBKG protein with skin and GI tissue (Supplementary Table S3), whereas TissueRanker identifies prostate tissue as being the highest co-expressed tissue (PCC = 0.75). Overall, 35% of the interactors in the complex were highly co-expressed (PCC > 0.8) and 70% were moderately or highly co-expressed (PCC > 0.5) with the IKBKG protein. This provides strong experimental support for the prediction, also detailed in our Discussion, despite its lack of explicit prostate annotation using our gold standard criteria. All these examples represent only a few of the ‘best’ and the ‘worst’, however, again with the average overall complexes performing highly accurately (overall AUC = 0.78). In general, proteins most specific to individual diseases and tissues, without diversity of phenotypes (such as LMNA discussed later), proved to be best predicted as illustrated later in the text.

### Tissue ranking of genes involved in distinct phenotypes

The *LMNA* gene is a component of the nuclear lamina and plays an important role in nuclear assembly, chromatin organization, nuclear membrane and telomere dynamics. The *LMNA* gene (and protein complex) provided an example associated both with different phenotypes and a large variety of affected tissues (22), such as dilated cardiomyopathy (19,23), Charcot-Marie-Tooth disease (20), muscular dystrophy(21) or Hutchinson-Gilford progeria syndrome (24) (for details please refer to Supplementary Text S1).These *LMNA-*associated diseases have in common that they all show a high diversity of affected tissues (mainly heart, muscle and skin) and distinct phenotypes.

The literature-based gold standard ranked tissues for the LMNA protein according to the disease, meaning that muscle and heart tissues were ranked first for cardiomyopathy, whereas skin and muscle tissues were ranked first for Charcot-Marie-Tooth disease. Our TissueRanker prediction, however, ranked the tissues for the LMNA protein according to the coordinated expression of the corresponding protein complex, meaning that for both diseases, skin and heart tissues were ranked first (which are tissues in which patients with Charcot-Marie-Tooth disease and cardiomyopathy develop phenotypes).

Within the set of predictions for the LMNA protein complex, skin was the highest co-expressed tissue with an average PCC value of 0.85. Overall, 40% of the interactors in the complex were highly co-expressed (PCC > 0.8) and 85% were moderately or highly co-expressed (PCC > 0.5) with the hub protein LMNA. This may suggest not only pleiotropy of phenotype, but also possibly tissue-specific subcomponents of the complex. TissueRanker identified heart tissue as the second highest co-expressed tissue with PCC value of 0.48, with 8% of the interactors as highly co-expressed (PCC > 0.8) and 50% as moderately or highly co-expressed (PCC > 0.5) with LMNA. Only few proteins overlapped in their coordinated expression between skin and heart, again indicating that both predictions may be correct, but that subcomponents of the complex function differentially across tissues (and thus diseases). Only skin and heart tissues were significantly more co-expressed than other tissues in the ranking for LMNA, and the complex shows that TissueRanker can provide distinct tissue predictions, with some specificity for individual interactors within complexes differing across tissues and disease phenotypes.

### Tissue ranking for polygenic diseases

Polygenic diseases caused by the combination of more than only one gene [e.g. diabetes (25)] were likewise amenable to prediction of affected tissues using this method. In our benchmark study, 12 proteins were associated with diabetes mellitus type 2 (see Table 1 and Supplementary Table S3). Diabetes affects mainly pancreas, muscle, adipose, ovary, brain, heart and kidney tissue (26–31). Our gold standard provided a ranking, with kidney and adipose tissue ranked on top (see Supplementary Table S3). Our TissueRanker predictions not only confirmed the gold standard tissues (kidney and adipose tissues), but also detected other affected tissues that play a role in diabetes that were not detected by the gold standard, such as muscle, brain, ovary and heart.

As another example, five proteins are associated with Leigh’s disease (see Table 1 and Supplementary Table S3), which is a neurometabolic disorder affecting mainly brain and muscle tissue (32,33). As expected, the gold standard ranked muscle and brain tissues as the most relevant tissues for this disease and our TissueRanker prediction could confirm these two tissue types in three of five proteins. However, for the DLD protein, our TissueRanker prediction could identify the heart tissue as being relevant. Indeed, Rutledge et al. (1982) have confirmed that hypertrophic cardiomyopathy is a frequent associated finding in Leigh’s disease (34).

## DISCUSSION

In this study, we developed TissueRanker, a predictive approach that determines the tissue specificity of a broad set of protein complexes based on global human gene expression. We analysed the tissue specificity of 3590 protein complexes and the properties of complexes specific to one or several of 36 given tissues. Our overall performance benchmark yielded an AUC of 0.78, in contrast with a simpler per-protein baseline of AUC = 0.59, which was particularly striking, as many of the tested protein complexes are likely to be incompletely annotated in our gold standard, and a random model (AUC = 0.52). We highlighted three specific biological examples in which TissueRanker correctly predicted the most affected disease tissue, including LEP as playing a role in hippocampal and hypothalamus tissues, the IGF2 as being involved in smooth muscle and brain tissue, and the inhibitor of nuclear factor kappa-light-chain-enhancer of activated B cells *IKK-γ* as being involved in prostate tissue, which are discussed later in additional detail.

As discussed above, previous studies aimed at associating coordinated expression with human disease etiology either investigated the structure of the interactome alone (6) or associated disease phenotypes with expression patterns across tissues (1,35–37) or within tissues (38). Few studies had further begun to integrate both the data types, the interactome and expression data, to analyse the interplay between transcript expression and protein interactions in human tissues without yet linking them to disease etiology (5). Here, we combined all three of these aspects–the human interactome, a large compendium of human expression data and tissue specificity of both expression and disease manifestation. These data together enabled a methodology to accurately predict the tissue specificity of protein complexes' roles in human disease.

In associating protein complexes with human tissues and disease, it is vital to consider the commonality and complexity of pleiotropic effects. This is true both biologically and when estimating TissueRanker's performance based on a literature-derived gold standard. Molecular mechanisms deregulated in disease are of course diverse and can affect many tissues, only some of which may be captured by current curation. The IKBKG protein serves as an example, which was only associated with ectodermal dysplasia (OMIM #300301) in the literature (see Supplementary Table S3). Further investigation of the protein based on predicted associations, however, supported an additional association with prostate cancer and tissue as discussed below.

In addition to sparsity within the gold standard, a similar limitation was reflected in the incompleteness of this dataset’s tissue expression coverage. Although this is the best global map of normal-tissue expression data to date, important tissues such as liver and pancreas remain missing. Complexes correlated (and thus highly ranked) within missing tissues should thus again be treated carefully before drawing a conclusion.

A first step we took to mitigate these effects was to consider groups of tissue/disease associations together. This proved beneficial both for the highest ranked tissue predictions per disease and for gold standard tissue/disease phenotypes. TissueRanker's underlying data source is ultimately expression data, subject to noise and technical artifacts as usual in high-throughput data. Therefore, we suggest that the user considers all top-ranked tissues, for example, the top three tissues, or all tissues that were ranked above a certain threshold. Further, tissue specificity does not mean that a protein complex is always involved in only one tissue, but instead in a selected number of tissues. In fact, this is rather the norm, as of the 3590 protein complexes considered in this study, only 31% are linked to one tissue only.

Second, in addition to our global performance assessment, we decomposed TissueRanker's prediction evaluation both into tissue-specific and protein complex-specific AUC values. Both these indicated that the methodology introduces far fewer incorrect predictions than expected by chance, but more importantly emphasize the range of performance within ‘easier’ and ‘difficult’ contexts. The latter was characterized by heterogeneity in cellular lineages and, importantly, incomplete annotations in our literature-based standard. For example, the IKBKG protein complex was the ‘worst’ performing complex owing to an unexpected prediction of prostate tissue involvement in disease. However, the *NF-κB* essential modulator is also known as *IKK-γ*, which is a protein that is encoded by the *IKBKG* gene and which is a subunit of the *IκB* kinase that activates *NF-κB* (39). Both genes are studied in cancer development and progression (40), and in particular in prostate cancer (12,13), indicating a deficiency in our gold standard rather than in TissueRanker's predictions.

**Figure 6.**
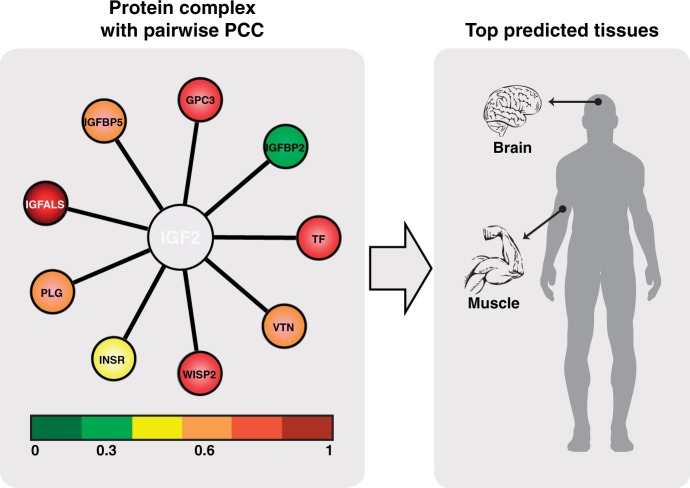
IGF2 as an example protein in which coordinated co-expression correctly predicts disease manifestation in brain and smooth muscle. IGF2 is a hub protein in a protein complex interacting with nine proteins (GPC3, IGFBP5, IGFALS, PLG, TF, INSR, IGFBP2, VTN and WISP2) as defined by the InWeb network. Pearson correlations between the hub protein IGF2 and its interactors are illustrated based on eight samples from smooth muscle tissue expression data under normal condition.

Together, this suggests that future studies may benefit by combining both additional literature-based gold standards and distinct expression maps, such as those of (7) and (4), to increase the number of tissues and replicates. This will expand the coverage of the recent and high quality expression map used here, which focused on biological replicates leading to a good tissue overlap with the gold standard, enabling an estimation of the performance of TissueRanker. Likewise, investigation of additional proteins not present in this gold standard may provide additional insight. For example, IGF2 is a hub protein interacting with nine other proteins in a protein complex defined by the InWeb network (see Figure 6). With no information provided in the gold standard itself, we predicted IGF2 as a highly coordinated expressed hub protein for smooth muscle and brain tissue (see Figure 6 and Supplementary Table S4), matching the finding of previous studies, highlighting *IGF2* central roles in muscle differentiation and cerebellum morphology (10,11).

A final important example is the *LEP* gene that was concordantly expressed in the palatine tonsile, hippocampal and hypothalamus tissues (see Supplementary Table S4). Leptin is principally secreted by white adipocytes, and plays a major role in the regulation of body weight. It signals nutritional status to specific hypothalamic nuclei to inhibit food intake or regulate energy expenditure to maintain constancy of the adipose mass. Therefore, *LEP* has mostly been studied in the context of fat tissue. The signaling role to the hypothalamus directly explains why the protein network around *LEP* is tightly regulated in that tissue. Sensory mechanisms of the mouth cavity are often overlooked when it comes to studies of food uptake and metabolism. Besides its muscle function, the tongue also has a sensory mechanism, which was suggested to play a direct role in modulating energy intake (41). The palatine tonsils, present in the back of the throat, function immunologically and provide additional sensory input to the glossopharyngeal nerve. No further literature is available on the regulation of *LEP* in the tonsils, but our findings would suggest further studies of *LEP* signaling in the oropharynx and locally within the central nervous system (42). In addition to food uptake regulation, it is emerging that *LEP* also regulates diverse aspects of hippocampal synaptic function that are thought to underlie learning and memory processes (8). Leptin-insensitive rodents display deficits in hippocampal long-term potentiation, long-term depression (9) and have impairments in spatial learning and memory tasks (43). Finally, in broader human disease, dysfunctions in the *LEP* system have recently been linked to neurodegenerative disorders such as Alzheimer’s disease, and therefore, *LEP* has been suggested as a novel therapeutic target in Alzheimer's disease (8).

In conclusion, we have presented new methods for the problem of detecting and quantifying genome-wide differences in the function of protein complexes among tissue types in human diseases. Importantly, this approach does not rely on disease literature or prior curation to establish tissue rankings, but works in a de novo manner to define these from gene expression data in normal tissues. The method is therefore expandable to any number of tissues when large-scale data from additional experiments are produced. At the same time, the method is well suited for the study and discovery of novel pleiotropic and multi-tissue roles of gene products. The human genome is relatively gene-poor, and it is likely that many pleiotropic effects still await discovery. Many of these effects might be hard to identify in a hypothesis-driven manner, and integration of high-throughput data, as in TissueRanker, may represent a more efficient approach going forward. The tissue-based target predictions can obviously go on to be further refined to more fine-grained, cell-type-specific phenotypes as increasingly detailed single cell expression data continue to become available.

## FUNDING

KUL PFV/10/016 SymBioSys, KUL GOA MaNet, Hercules III PacBio RS and FP7-HEALTH CheartED (to D.B. and Y.M.); The European Science Foundation (ESF) for the activity entitled ‘Frontiers of Functional Genomics’ [Exchange Grant #2939 to D.B.]; The Danish Council for Independent Research Medical Sciences (FSS) (to T.P.); a Postdoctoral Fellow of the Research Foundation - Flanders (FWO) (to L.T.); NSF CAREER [DBI-1053486 to C.H.]. Funding for open access charge: FP7-HEALTH SyBoSS [contract no.: 242129].

*Conflict of interest statement*. None declared.

## Supplementary Material

Supplementary Data
