# Supplementary material for: Concordance of gene expression in human protein complexes reveals tissue specificity and pathology
Source: Nucleic Acids Res. 2013 Aug 5;41(18):e171. doi: 10.1093/nar/gkt661 (PMC3794609; doi:10.1093/nar/gkt661)
Supplement: Supplementary Data [file supp_41_18_e171__index.html]

Concordance of gene expression in human protein complexes reveals tissue specificity and pathology — Concordance of gene expression in human protein complexes reveals tissue specificity and pathology — Supplementary Data 

# Concordance of gene expression in human protein complexes reveals tissue specificity and pathology

## 

files

**Files in this Data Supplement:**

- Supplementary Data - pdf file
- Supplementary Data - xlsx file
