# Supplementary material for: Concordance of gene expression in human protein complexes reveals tissue specificity and pathology
Source: Nucleic Acids Res. 2013 Aug 5;41(18):e171. doi: 10.1093/nar/gkt661 (PMC3794609; doi:10.1093/nar/gkt661)

Supplementary Material

Text T1: Description of the diseases in the from tissue ranking of genes involved in distinct phenotypes

Dilated cardiomyopathy is a disorder characterized by ventricular dilation and impaired systolic function, resulting in congestive heart failure and arrhythmia (OMIM #115200). Cardiomyopathy dilated with hypergonadotropic hypogonadism (OMIM #212112) is characterized by the association of dilated cardiomyopathy, genital anomalies, hypergonadotropic hypogonadism, and other variable clinical manifestations including scleroderma-like skin, graying and thinning of hair. Limb-girdle muscular dystrophy (OMIM #159001) is an autosomal dominant degenerative myopathy with age-related atrioventricular cardiac conduction disturbances, dilated cardiomyopathy, and the absence of early contractures and is characterized by slowly progressive skeletal muscle weakness. The related Emery-Dreifuss muscular dystrophy (OMIM #181350) is a degenerative myopathy characterized by weakness and atrophy of muscle without involvement of the nervous system, early contractures of the elbows, Achilles tendons and spine, and cardiomyopathy associated with cardiac conduction defects. The Charcot-Marie-Tooth disease (OMIM #605580) is the most common inherited disorder of the peripheral nervous system and is associated with neuropathy which is characterized by signs of axonal regeneration in the absence of obvious myelin alterations, normal or slightly reduced nerve conduction velocities, and progressive distal muscle weakness. In some cases the skin was severely involved [26]. The Hutchinson-Gilford progeria syndrome (OMIM #176670) is a rare genetic disorder characterized by features reminiscent of marked premature aging.

Figure S1: Based on the genome-wide expression data, we computed the pairwise correlation (PCC) between tissues, producing tissue distance matrices containing all the pairwise distances for a given set of tissues. Here, the distance matrices are visualized as heatmaps of all tissues from our study (A) and the 14 benchmark tissue categories (B), respectively.

A

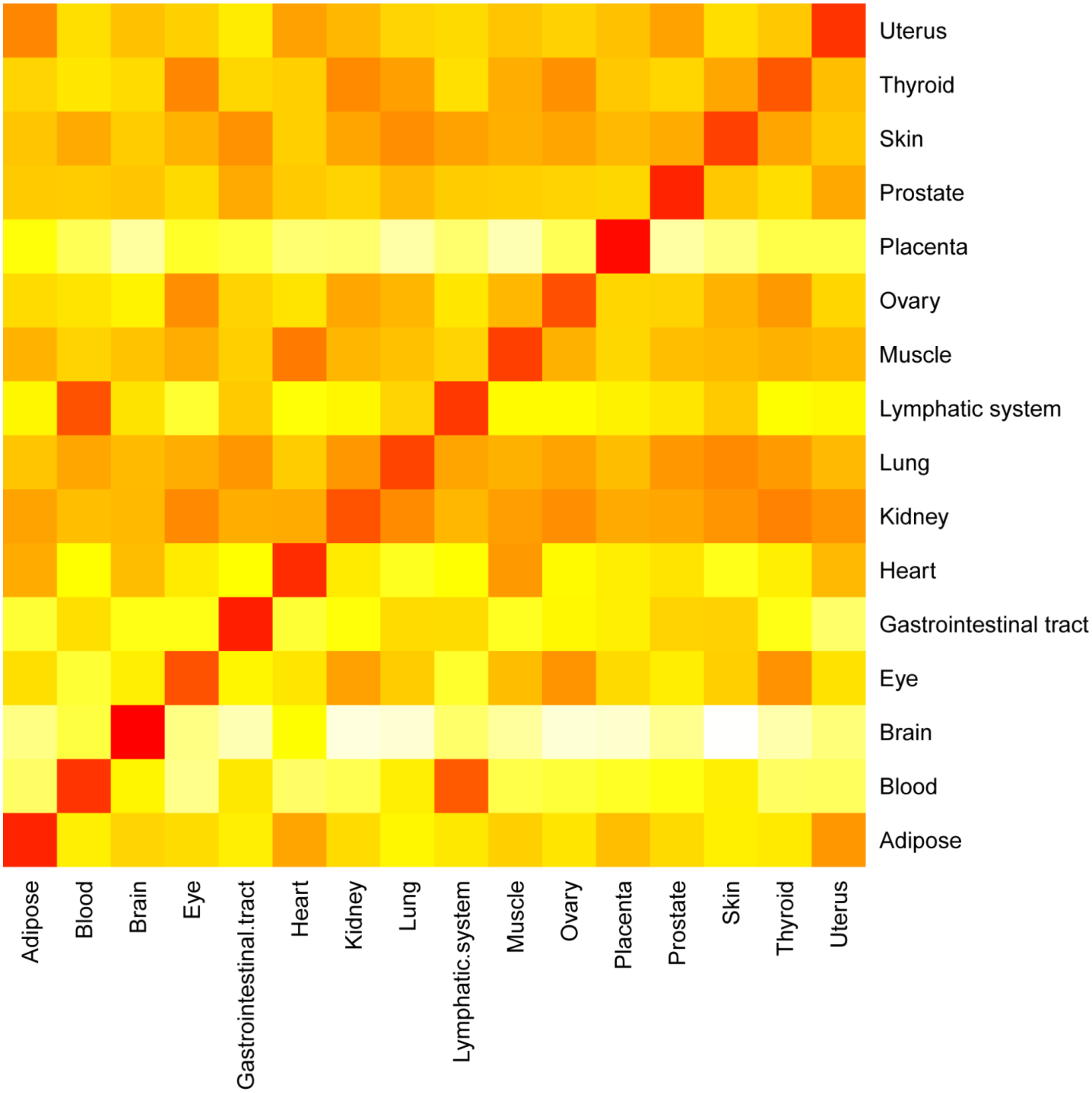

B

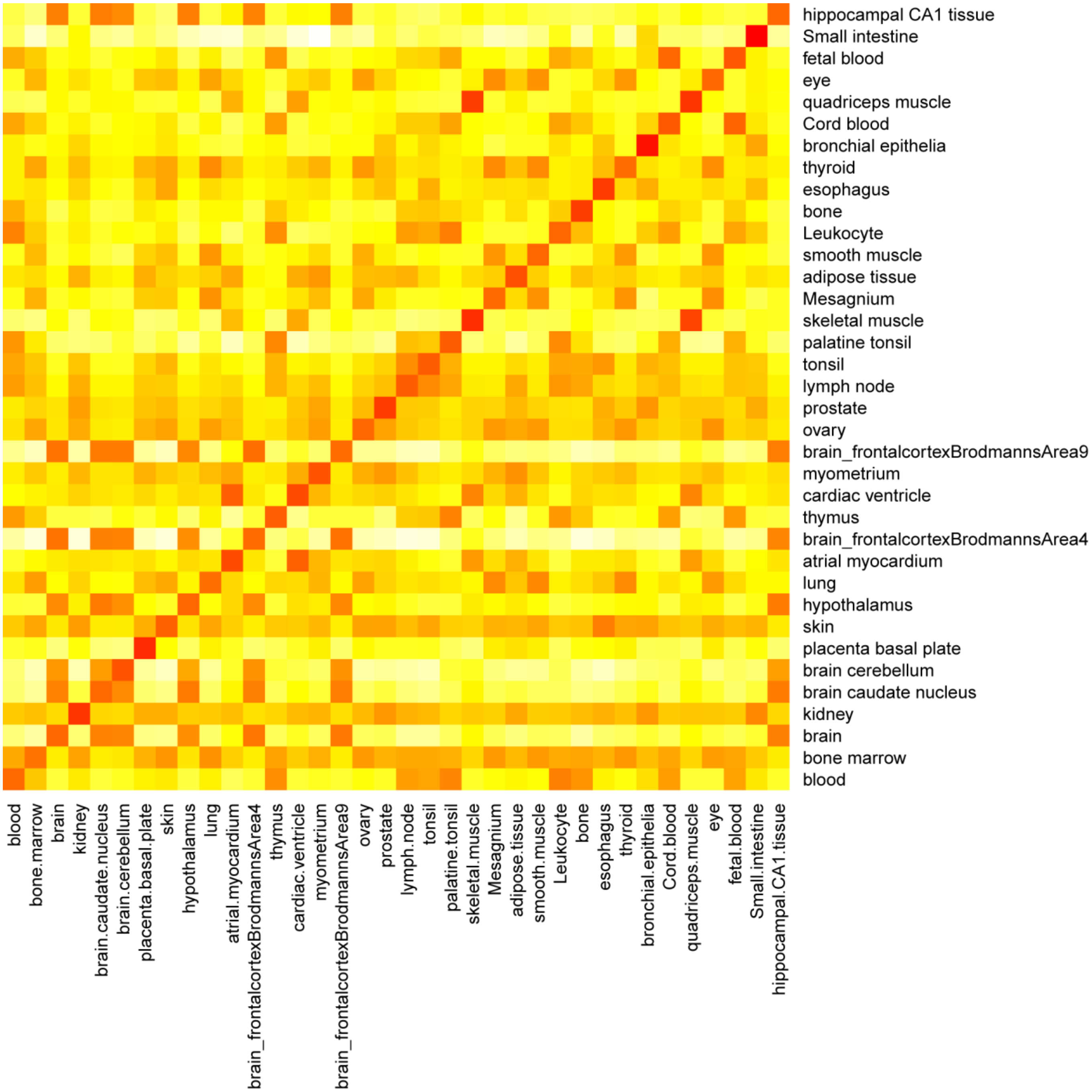

hippocampal CA1 tissue
Small intestine
fetal blood
eye
quadriceps muscle
Cord blood
bronchial epithelia
thyroid
esophagus
bone
Leukocyte
smooth muscle
adipose tissue
Mesagnium
skeletal muscle
palatine tonsil
tonsil
lymph node
prostate
ovary
brain_frontalcortexBrodmannsArea9
myometrium
cardiac ventricle
thymus
brain_frontalcortexBrodmannsArea4
atrial myocardium
lung
hypothalamus
skin
placenta basal plate
brain cerebellum
brain caudate nucleus
kidney
brain
bone marrow
blood
blood
bone.marrow
brain
kidney
brain.caudate.nucleus
brain.cerebellum
placenta.basal.plate
skin
hypothalamus
lung
atrial.myocardium
brain_frontalcortexBrodmannsArea4
thymus
cardiac.ventricle
myometrium
brain_frontalcortexBrodmannsArea9
ovary
prostate
lymph.node
tonsil
palatine.tonsil
skeletal.muscle
Mesagnium
adipose.tissue
smooth.muscle
Leukocyte
bone
esophagus
thyroid
bronchial.epithelia
Cord.blood
quadriceps.muscle
eye
fetal.blood
Small.intestine
hippocampal.CA1.tissue

Figure S2: The distribution of the complex specific AUC values for all benchmark protein complexes in this study, estimating the FP rates based on a comparison of the ranking of the gold standard (Expression Ranking) with the ranking from our TissueRanker prediction and the Random Model. The corresponding AUC values can be found in Supplementary Table S5.

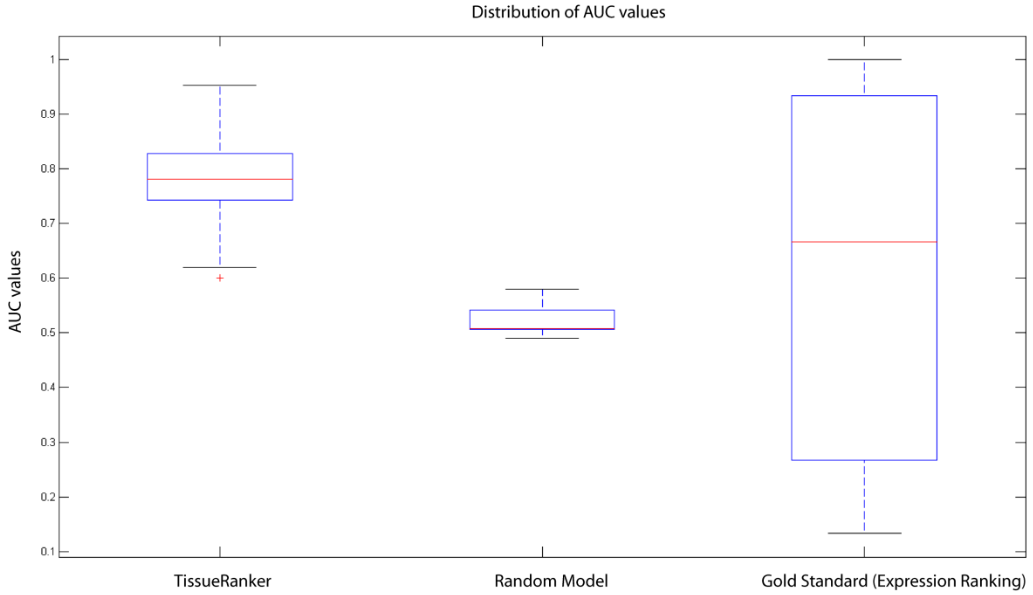

Figure S3: Scatter plot between the AUC per tissue and the number of sample per tissue shows that tissues that were more difficult to predict were characterized by a high-diversity of included cell types (*e.g.,* blood, Lymphatic system, or brain tissue), whereas well-characterized tissues were typically more homogeneous.

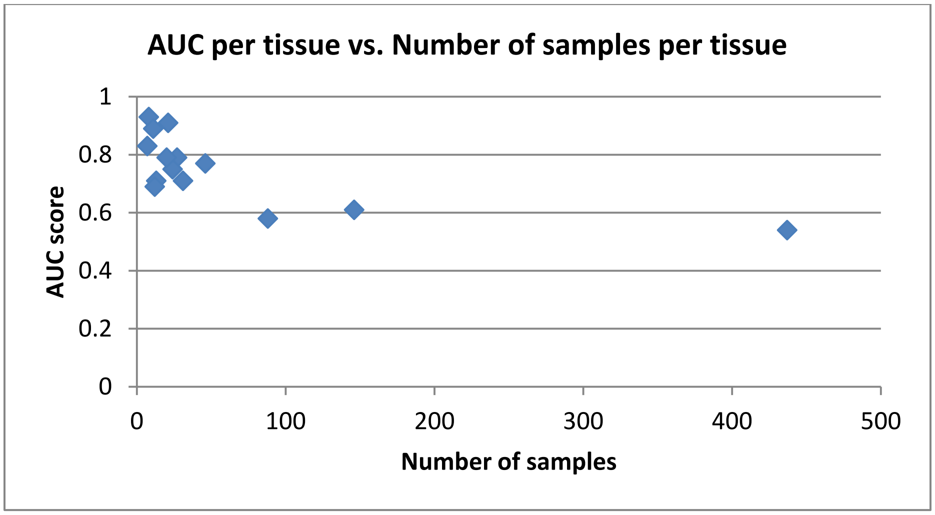

Figure S4: Scatter plot between the AUC per complex and the complex size (number of proteins per complex) shows that the size of the size had not impact on the performance (AUC) of its corresponding protein complex.

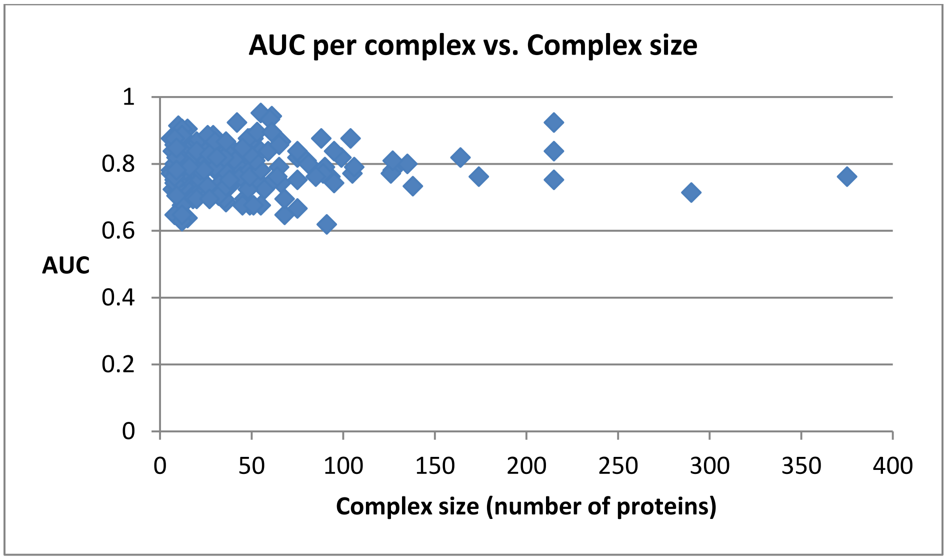

Figure S5: Scatter plot between the AUC per complex and the number of diseases that are assigned to each complex in the benchmark shows that the number of diseases had not impact on the performance (AUC) of its corresponding protein complex.

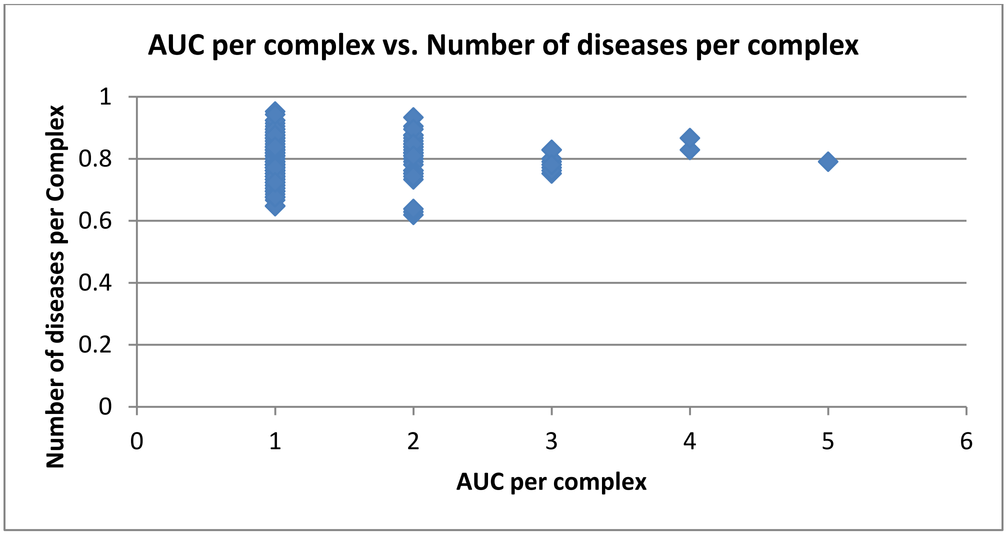

Supplement: Supplementary Data [file supp_gkt661_nar-01095-met-n-2013-File003.pdf]
